# Efficacy of an Emergency Department-based Motivational Teenage Smoking Intervention

**Published:** 2006-12-15

**Authors:** Kimberly Horn, Geri Dino, Candice Hamilton, N Noerachmanto

**Affiliations:** West Virginia University, Department of Community Medicine, Prevention Research Center, Office of Drug Abuse Intervention Studies; Department of Community Medicine, West Virginia University, Morgantown, WVa; Department of Community Medicine, West Virginia University, Morgantown, WVa; Department of Community Medicine, West Virginia University, Morgantown, WVa

## Abstract

**Introduction:**

Motivational interviewing techniques have been minimally researched as a function of a teenage smoking intervention. The present study examined the efficacy of a theory-based motivational tobacco intervention (MTI).

**Methods:**

A randomized two-group design was used to compare 6-month post-baseline quit and reduction rates among teenagers who received the MTI with those who received brief advice or care as usual. Participants were smokers aged 14 to 19 years (N = 75) who presented for treatment in a university-affiliated hospital emergency department (ED). Motivational interviewing techniques were used by trained providers to facilitate individual change; stage-based take-home materials also were provided.

**Results:**

Similar to past clinic-based studies of motivational interviewing with teenage smokers, our study found negative results in terms of intervention efficacy for cessation. Six-month follow-up cessation rates were nonsignificant — two teenagers quit smoking. Among teenagers who were available at follow-up, a medium effect size (Cohen's h = .38) was found for reduction and a large effect size (Cohen's h = .69) was found for percentage reduction, although these results also were not statistically significant.

**Conclusion:**

Although the major findings of this study were not significant, the reductions in tobacco use suggest that motivational interviewing may be a clinically relevant counseling model for use in teenage smoking interventions. However, many questions remain, and the current literature lacks studies on trials with significant outcomes using motivational interviewing in smoking cessation. Additionally, more research is needed to examine the suitability of the ED for MTI-type interventions.

## Introduction

Evidence supporting the long-term health consequences of teenage smoking is mounting. As a result, researchers and clinicians are beginning to implement a variety of intervention approaches for teenagers who smoke and want to quit. The most commonly used approach is to implement school-based group intervention programs (1), but other approaches include clinic-based individual interventions, family-based programs, and Internet self-help programs (1,2). Although there is sound research supporting the efficacy of school-based programs such as Not On Tobacco (N-O-T) (3) and Project EX (4), there is limited research on the feasibility and efficacy of other intervention approaches for youth. Thus, many questions about optimal approaches remain unanswered (5). Although schools are critical venues for youth tobacco control, it is unrealistic to assume that school-based interventions can serve, and be suitable for, millions of U.S. teenage smokers. Focusing only on schools limits access to intervention, particularly for high-risk teenagers who attend school infrequently, hold negative attitudes toward school, have dropped out, are detained, or attend schools with limited resources. Ideally, youths who smoke should be saturated with options for cessation in multiple settings — schools; churches; primary care settings; and other clinical settings, including hospital emergency departments (EDs).

One minimally researched approach for teenage smoking is a clinic-based intervention that incorporates motivational interviewing (6). Motivational interviewing is a counseling technique used to promote behavior change that 1) can be designed in developmentally appropriate ways, 2) can be tailored to individual needs, 3) is flexible and brief, 4) can be combined with additional components (e.g., self-help materials), 5) can reinforce efforts made in schools and communities, and 6) can address individual levels of motivation and confidence for quitting (6). Based in client-centered therapy, social cognitive theory, and cognitive behavioral therapy, motivational interviewing incorporates six elements (FRAMES): feedback, responsibility, advice, menu of strategies, empathy, and self-efficacy (7). Goal setting, follow-up, and timing also are important aspects of motivational interviewing (8). This intervention technique assumes that the responsibility for change rests within the patient. Importantly, interventionists create conditions that promote motivation and confidence to change. This is achieved using five basic strategies: 1) asking open-ended questions, 2) using reflective listening, 3) developing discrepancy (i.e., helping the patient understand the differences between the present behavior and his or her values), 4) providing personalized feedback, and 5) eliciting self-motivating statements (6). Providing tailored and personalized feedback to patients is intended to help them understand the relationships between their unhealthy behaviors (e.g., smoking) and personal consequences. Motivational interviewing also addresses high dropout rates (9) encountered by some multisession interventions; program dropout has been associated with motivational ambivalence (6). Motivational interviewing is intended to influence the ambivalence that teenagers have about changing their smoking behaviors using a single “on-the-spot” interaction that is nonconfrontational and empathic (9). Experts recommend using motivational interventions in health care settings in which patients who smoke may receive services they might not otherwise receive (10). Moreover, motivational interventions are well suited for health care settings because they are brief (i.e., usually less than 30 minutes). Recent studies show that motivational interventions are at least as effective as other treatment methods for mild-to-moderate alcohol and tobacco problems and are clearly superior to no treatment among adults (6,7,9-11). However, despite the purported benefits of motivational interviewing, only a handful of studies have found it suitable for use with adolescents (10,12,13).

The present study examined the efficacy of a theory-based motivational tobacco intervention (MTI)The present study examined the efficacy of a theory-based motivational tobacco intervention (MTI) for smokers aged 14 to 19 years who presented for treatment in a hospital ED in Morgantown, WVa. The ED was selected as the target health care setting because 1) it is often used as a source of primary health care and 2) the often long waiting period is conducive to supplemental services. Efficacy was examined by comparing the overall reductions in smoking among teenagers randomly assigned to MTI or brief advice (BA). We hypothesized that the MTI smoking quit and reduction rates would be significantly higher than those of the BA group. State-of-the-art motivational interviewing methods, the positive and negative study outcomes, and implications for harm reduction among teenage smokers within a clinical health care setting are discussed.

## Methods

### Participants

Participants were patients aged 14 to 19 years presenting for care for any reason in a suburban, university-affiliated hospital ED between January 2002 and September 2004. Patients were eligible if they 1) reported smoking on 1 or more days in the past 30 days, 2) volunteered to participate, and 3) provided written assent and consent (a parent or guardian had to be present). Patients were ineligible if they 1) arrived in police custody; 2) had communication deficits, such as an inability to speak English, or were severely hearing-, vision-, or speech-impaired; 3) were deemed mentally incompetent; 4) had life- or limb-threatening conditions (i.e., acuity); or 5) were verbally or physically combative.

### Procedure

A randomized two-group design was used to compare participants who received either the MTI or BA intervention. Following the recommendations of Miller and Rollnick (6) and Carroll et al (i.e., Project Match) (14), the MTI consisted of 1) screening; 2) a 15- to 30-minute patient-tailored face-to-face motivational interview including a readiness assessment, a reflection on smoking behaviors, and a health inventory; 3) a stage-matched, self-help take-home workbook with audio (i.e., the Power Guide) (15); 4) one handwritten personal postcard within 3 days of the ED visit; and 5) three follow-up “booster” phone calls at 1, 3, and 6 months post-ED visit. Representing standard care, the BA intervention consisted of 1) screening; 2) no more than 2 minutes of generic advice to quit smoking; 3) referral to Health Line, the state 1-800 telephone help information line, a general information source (16); and 4) one follow-up phone call 6 months post-ED visit.

The intervention providers employed for this study had relevant backgrounds in social work, psychology, and public health education. Before starting the study, providers received approximately 75 hours of training on motivational interviewing strategies, the study protocol, and all relevant study forms. Training, conducted by the researchers, included role playing, hands-on practice, and direct observation of providers and feedback in the ED.

A total of four intervention providers were located in the ED during the busiest patient intake periods: 12:00 pm until 12:00 am, Monday through Friday. Providers initially approached patients while they were in the ED waiting area, following check-in. Before approaching a patient, the providers drew from a single pile of intervention folders (containing all necessary forms for protocol completion), which were located in a secure ED area. The folders were sequentially numbered in a single pile as sorted by the SAS (SAS Institute, Inc, Cary, NC) random number function. Each randomized manila folder contained either the MTI or the BA protocol set of equal size and weight. Each provider was blinded during the initial screening and did not know to which group the participant was assigned until the folder was opened after the screening was complete. Study forms are discussed next in the context of the intervention because they were used for both data collection and as intervention aids for personalized feedback. Extensive details are provided here on intervention methodology not typically reported in the literature.

#### Screening

Teenagers were initially approached during the waiting period before ED treatment. Typically, the entire screening and intervention process was completed before a patient's contact with a physician. Consistent with motivational interviewing techniques, a necessary first step was to engage patients in the identification of the problem behavior — in this case, smoking; as such, teenagers were first screened by providers to ascertain smoking status. Teenagers who reported smoking on 1 or more days in the past 30 days were briefed on the study, and consent and assent were obtained. Initial data were collected using an individual information form that documented the beginning time of screening and general patient demographics. Next, a screening and general assessment form documented teenagers' baseline characteristics, including factors such as smoking rates and previous quit attempts. Nicotine dependence also was assessed using the Fagerstrom Tolerance Questionnaire (FTQ) (17). FTQ is a widely used and reliable 8-item scale for adults; minor modifications were made for use with teenagers (17-19). An aggregate score between 0 and 2 indicated very low dependence, 3 or 4 indicated low dependence, 5 indicated medium dependence, 6 or 7 indicated high dependence, and 8 to 11 indicated very high dependence. Last, a carbon monoxide (CO) record was used to document the results of a CO test administered to validate baseline self-reported smoking status. The CO test was a brief breath test in which patients exhaled into a small disposable tube attached to a digital CO monitor that indicated the patients' CO levels; a CO score of less than 9 ppm confirmed a patient's self-reported nonsmoker status. This test enhanced confidence in the accuracy of the self-report measures (20,21). At the time of the study, we chose CO instead of saliva cotinine to validate smoking status because it was lower in cost, easier to obtain, did not require obtaining and storing bodily fluids (21), and was less threatening to individuals concerned about drug testing (22). Studies suggest that there is little advantage of using costly cotinine samples over CO samples (20) and that CO is a viable alternative to cotinine (21). Information about the patient's smoking history was used later to provide personalized feedback to MTI participants; providing such information is important for motivational techniques. Upon completion of the screening, providers broke the seal on the intervention packet folder and patients were administered either the MTI or BA intervention as indicated. Each intervention arm is described next. CO data were not collected at the 6-month follow-up because final contact was made by telephone.

#### Components of MTI treatment

##### Readiness assessment. 

Readiness was assessed using two items that guided the MTI provider through the first stage of intervention — determining patient readiness to quit smoking. A fundamental principle of motivational interviewing is to foster patients' motivation and confidence to change. Thus, this part of the intervention gave providers the opportunity to assess patients' confidence and motivation to quit smoking (both were measured using a questionnaire with a 10-point Likert scale ranging from 1 = not at all to 10 = completely). A provider used patient responses to probe further by asking questions such as, "What would need to happen to move you from a 1 to a 6 or a 10?" The questionnaire also assessed the patient's current stage of change (6) (ranging from 1 = do not plan to quit smoking in the next 6 months to 4 = have made serious quit attempt in the past 6 months). Understanding reasons for smoking was important to future processes; the patient responses were critical in determining the sequence and emphasis of the intervention to establish a foundation for continued intervention and personalized feedback (23-25).

##### Reflection. 

This technique facilitated patients’ reflection on smoking behavior; moreover, it allowed providers the opportunity to engage in reflective listening — an essential element of motivational interviewing. Providers queried patients about their 1) reasons for smoking (23), 2) common smoking places or situations, 3) smoking history, 4) frequency of smoking among family and friends, and 5) anticipated support among family and friends. Provider questions and queries were intended to heighten patients’ awareness of the “who, where, and when” of their smoking. For instance, a provider might say: “You said that you typically smoke at your best friend’s house. . . . Do you have some ideas about other places you and your friend can hang out when you try to quit smoking?” Important for motivational interviewing, these reflective strategies also were important for developing discrepancy; open-ended questions were intended to help patients identify the perceived benefits as well as the negative aspects of their smoking behaviors (23-25. An example of this type of question would be, “How might smoking hinder your dating life?”

##### Health inventory. 

Health inventory was designed to explore patients' smoking behavior and the potential physical, social, and emotional consequences of smoking. Providers used this information to tailor discussions about the adverse consequences of smoking and relate smoking to reported complaints (e.g., asthma, bronchitis, colds, excessive phlegm). Again, the intent of this strategy was to build discrepancy, particularly related to the personal consequences of smoking. At this point in the intervention, providers worked to elicit patients' self-motivating statements for change (26).

Overall, based on patients' responses during the three intervention components described above, smokers were advised to quit smoking using menus of strategies recommended by Miller and Rollnick (6). Specifically, MTI included patient-tailored personalized feedback on 13 potential topics. Key topics were confidence building, including support and encouragement; feedback on screening and assessment results, including discussion of how their smoking compared with that of the general teenage population (i.e., normative information); discussion of the consequences of smoking, both past and potential; recommendations to reduce or abstain from smoking along with help in making that decision; and goal setting. For example, some teenagers were highly motivated to quit smoking but had low confidence that they could succeed (27). Others had low motivation and high confidence. The basic goal was to help patients identify arguments for change (motivation) and attainable steps for quitting (confidence). Examples of probing questions related to building motivation included: "What do you like about smoking?"; "What do you dislike about smoking?"; and, after the interventionist summarized the patient's dilemma, "Where does that leave you now?" A strategy for dealing with low confidence was to ask: "What is your biggest worry about quitting smoking?" By determining a patient's main concern about quitting, the provider and patient brainstormed meaningful solutions. A final step involved determining whether the patient was ready to set a goal (e.g., quitting, reducing). Information was also tailored to the sex of the patient. Thus, a provider might say to a female patient: "You said you smoke when you feel down. . . . Sometimes girls struggle with this. . . . I have some tips that might help you. . . . Would you like to hear them?"

##### Self-help audio workbook. 

All MTI participants were provided with the Power Guide, a gender-sensitive, stage-tailored, self-help workbook. Consistent with the recommendation of Miller and Rollnick (6) to include adjunctive motivational materials as a function of motivational enhancement, patients were asked to take home the workbook to support their on-your-own quit efforts during the following 10 weeks. The workbook, based on the American Lung Association's N-O-T program (a Substance Abuse and Mental Health Services Administration [SAMHSA] Model Program) (3,28), was intended to build on the motivational interviewing received in the ED and was created by the same developers of the N-O-T program. Similar to the N-O-T curriculum and research on self-help (29-31), the workbook incorporated reasons for smoking, excuses for not quitting, the realities of smoking, motivation and confidence building, addiction processes, self-management and stimulus control, coping with trigger situations, social skills and social influences, cognitive and behavioral restructuring, relapse prevention, nicotine withdrawal, weight management, and family and peer pressure (32). Before ED departure, providers instructed the patient on how to use the workbook in general and according to the patient's stage of readiness to quit smoking. For example, teenagers who were thinking about quitting were instructed to follow a sequence of activities intended to build motivation and confidence and move them closer to action. To accommodate a variety of reading levels among teenagers, the workbooks included a narrated audio CD.

#### Components of BA treatment

The BA intervention was developed according to ED care as usual for teenage smokers (10). By design, it was intended to be shorter in duration than the MTI. Specifically, BA patients received not more than 2 minutes of generic advice to quit smoking and a referral card to the state Health Line while in the ED. Health Line is a statewide 24-hour-per-day, 7-day-per-week service offering 1-800 telephone access to information specialists. It provides general health information or advice, and referrals. Health Line is a part of the hospital's standard referral practice. A teenager who called Health Line to ask about smoking cessation was referred to a pediatrician or the local cancer society per protocol.

#### Follow-up procedures for MTI and BA

Follow-up phone calls from research staff (different than the provider for a given patient) were made to MTI patients at 1, 3, and 6 months post-baseline. MTI intervention participants also received a handwritten postcard from providers within 3 days of their ED intervention. BA participants were contacted at 6-month follow-up only, as the 1- and 3-month follow-up calls were considered "boosters" for the MTI group. The critical comparison point for quit and reduction rates was at the 6-month follow-up.

### Data analysis

To assess baseline differences, we identified variables that could be related to quitting or reduction among youth. These included age, grade level, nicotine dependence, number of cigarettes smoked per day on weekdays and weekends, and number of previous quit attempts. Because sex is an important exploratory variable, analyses were conducted overall and separately for male and female participants. Because the comparisons involved multiple *χ* ^2^ and *t*-test analyses, the correction for controlling the heightened error was applied by dividing the level of significance (.05) by 10 (.005) (33).

A patient was considered to be a nonsmoker if self-reported quitting was indicated at telephone follow-up (i.e., a yes response to the question, "Have you quit smoking?"). Data on days of continuous abstinence were also collected. Chi-square analyses were used to calculate both intent-to-treat (total participants available at follow-up who quit divided by the full sample) and compliant sample quit rates (total participants available at follow-up who quit divided by the available sample). Compliant sample analysis was used to assess the relative efficacy of MTI vs BA. Intent-to-treat was used to assess the MTI intervention efficacy independently. A participant was considered a reducer if he or she reported smoking fewer cigarettes at follow-up than at baseline. Reduction rates from baseline were calculated, as were mean percentage rates among teenagers who reduced from baseline.

An attrition analysis was conducted to identify any baseline differences between teenagers who provided 6-month follow-up data and those who did not. A two (present/absent) by two (MTI/BA) MANOVA on the factors of number of cigarettes smoked on weekdays and weekends, nicotine dependence, age, and previous quit attempts was performed.

## Results

### Participant Characteristics

A total of 128 patients were eligible for study participation; 76 (59.4%) were initially enrolled. One participant was discharged before finishing the assessment, leaving a baseline sample of 75. Among the participants, 43 (57.3%) were female and 72 (96.0%) were white (Table 1). The mean age of the study participants was 17.8 years; 41 (54.7%) patients were randomized into the MTI group. One teenager withdrew from the study following the MTI assessment, bringing the final sample to 74. Among those who chose not to participate (n = 52), the most frequently cited reason for refusal was acuity (53.8%); one third of patients offered no reason for refusal (32.7%) (data not shown).

### Baseline comparisons

Participants were equivalent on most baseline variables based on the corrected *P* value of .005. The only significant difference was in number of cigarettes smoked during weekends. Complete baseline participant profiles are shown in Table 1 and Table 2.

### Quit rates

Comparative analysis between MTI and BA quit rates at 6 months post-baseline was not statistically significant (Table 3). One participant in each group reported quitting at 6-month follow-up. Sex analysis was not applied because of the low number of quitters. No significant differences between absent and present teenagers at 6-month follow up were observed.

### Reduction rates

MTI patients showed greater reduction than BA patients (Table 4). Comparison of 6-month post-baseline values for MTI and BA participants was not significant, but a medium effect size was found for the compliant subsample (Cohen's h = .38); a small effect size was found for the intent-to-treat subsample (h = .15). Among teenagers who reduced, MTI teenagers reduced more than BA teenagers. The 6-month follow-up comparison revealed a large effect size (Cohen's h = .69) for mean percentage reduction (Table 5).

In total, MTI patients received a mean of 30.6 minutes of provider contact (SD = 4.9) during the course of intervention, including 1-, 3-, and 6-month follow up (data not shown). BA patients received a mean of 11.9 minutes of provider contact (SD = 6.6), including 6-month follow-up contact. More than half (56.3%, 9/16) of the MTI patients who participated in the 6-month follow-up reported using the Power Guide. Responses to the workbook were generally positive: 88.9% (8/9) of those who used it said they would recommend it to a friend; 77.8% (7/9) said that the workbook helped them change their smoking behavior. There was no significant relationship between smoking reduction and workbook usage.

## Discussion

### Efficacy

Comparable at baseline, MTI and BA patients generally smoked about one half pack of cigarettes per day and were low nicotine dependent. Interestingly, this ED sample differed from the school-based samples of the same cohort enrolled in the investigators' other cessation studies (4,18). Specifically, the school-based samples smoked a pack of cigarettes per day, were medium to high nicotine dependent, and were almost 2 years younger. Differences suggest that the clinic-based teenage smoker population may present a different kind of smoker than that observed in school-based studies. Further testing of clinic-based MTI may require refining strategies tailored toward an older but less addicted smoker.

Despite use of methods recommended by experts (6-10), our study found limited cessation effects. These findings are similar to other ED-based studies using motivational interviewing with teenagers. In one of the few published studies of motivational interviewing among teenage smokers, Colby and colleagues also conducted an ED-based brief intervention (10). Similar to our study, Colby randomized teenagers (n = 40) into either a 30-minute motivational interview or a 5-minute recommendation to quit smoking. Among patients receiving a motivational intervention, 20% were smoke free, compared with 10% in the comparison group. Although there was a small to medium effect size (Cohen's h = .28), the difference was not significant. Approximately 95% of patients in the Colby study returned for 3-month follow-up.

The current study revealed that overall reduction rates were two times greater among MTI than BA participants. Although the difference was not significant, MTI patients reduced use as much as 60%, and a large effect size was found. Other studies of motivational interviewing have found reductions rather than complete abstinence in the targeted behaviors. Woodland and colleagues conducted a study of ED patients aged 18 to 19 years treated for an alcohol-related event (34). Patients were randomized to standard care or to one session of motivational interviewing. At 6-month follow-up, the motivational group had a significantly lower incidence of drinking and driving, traffic violations, and alcohol-related problems and injuries than those in standard care. In a second study, ED patients aged 13 to 17 years were randomized to the same two treatment conditions (35,36). In both studies, results for reduced drinking rates were not significantly different between interventions but indicated a main effect for drinking reductions. Results from our trial and other recent trials using motivational interventions with adolescents indicate that motivational approaches may result in decreases rather than cessation of the targeted health behavior (e.g., tobacco use). Given these findings, motivational enhancement may be considered as a harm reduction approach (37). In this context, reduced exposure to smoking and its by-products may be important for smokers who are unable to quit or who are actively trying to quit (38). For example, Hatsukami et al found that adult smokers who reduced cigarette use showed significant reductions in lung carcinogen metabolites, suggesting that decreases in carcinogenic uptake can be clinically meaningful (38). Moreover, reduction of tobacco use has been considered by some experts as a potential transitional goal toward cessation. As pointed out by O'Leary-Tevyaw and Monti, motivational enhancement may be most effective with excessive behaviors, such as tobacco or other drug addiction (37). In the present study, the sample included light (one half pack per day), low-nicotine–dependent smokers. Without a doubt, reduced smoking as a harm-reduction approach is not a substitute for complete cessation. Quitting smoking is the only known way to reduce tobacco-related mortality and morbidity. Further research is needed to determine if motivational interviewing may provide opportunities for harm reduction as individuals move toward complete cessation.

### Challenges and limitations

Our most critical study barrier was recruitment, particularly related to patient acuity. Almost half of patients who refused to participate did so because of acuity, which was the most cited reason for patient refusal. According to our hospital data, about one fourth of ED patients between the ages of 14 and 18 years are routinely admitted to the hospital for further treatment. Pain, discomfort, and illness severity is largely a subjective experience of the individual. However, even among patients who did not require hospitalization, many reported physical discomfort or emotional stress that hindered their willingness to participate in the study. Beyond physical acuity, many teenagers (27.1%) presented with psychiatric problems, and our providers never approached them.

Obtaining consent and assent for younger teenagers was another study challenge. During the study, 74.7% of the study patients were aged 18 to 19 years, even though only 56.1% of the total age-eligible patients in the ED were between the ages of 18 and 19 years. Only 12.0% of our study patients were younger than 17 years of age. This disproportionate percentage suggests that certain aspects of the process for consenting minors make patient recruitment more difficult in younger patients. This may in part be a result of younger teenagers not wanting their parents to know they smoke. Older teenagers may be less concerned about parental consequences. This is an important consideration for ED-based interventions because a parent or guardian must consent for treatment of a minor. Another limitation is that the majority of study participants were white. Larger, more diverse samples from multiple settings are necessary to make further generalizations about the efficacy and utility of MTI and other motivational interventions. Also, follow-up found low retention rates, presenting potential biases in our data. However, it is important to note that the attrition analyses found no significant differences between teenagers who were absent at 6-month follow-up compared with those who were present.

Motivational approaches are widely accepted in the literature and recommended as potential alcohol, tobacco, and other drug use interventions. However, this approach has been minimally researched as a teenage smoking intervention. Similar to other studies, we found notable reduction in smoking behavior — two times more MTI than BA patients reduced smoking — but no significant differences in cessation. In spite of recommendations to use motivational interviewing methods with teenage smokers, many questions remain, and the current literature lacks trials with significant outcomes in smoking cessation. Motivational interventions have gained attention in the field because, among adults, they have the highest effect sizes among all treatments for alcohol abuse and dependence (38-40). Other research shows that motivational interviewing offers cost-effective alternatives to traditional, longer-term treatments with comparable outcomes (14). However, the evidence for adolescents is not as solid, and more research is needed to answer numerous questions. Our conclusion is not to suggest that MTI and other motivational interventions are ineffective. Instead, we assert that the current study revealed reductions in tobacco use and lends itself to further study as a harm-reduction model of intervention for teenage smoking. Moreover, more research is needed to examine the suitability of the ED for MTI-type interventions. In particular, the field requires greater understanding of the type of teenage smoker that presents in clinical settings such as the ED, the unique barriers of acuity, and the imminent presence of parents or guardians during the intervention process.

## Figures and Tables

**Table 1:** Demographic Characteristics and Baseline Comparisons, by Intervention Group, Emergency Department-based Motivational Teenage Smoking Intervention, Morgantown, WVa, 2002–2004

**Characteristics**	**Total Sample^a^ (N = 75) No. (%)**	**MTI Group (n = 41) No. (%)**	**BA Group (n = 34) No. (%)**	***P* b**
**Sex**				
Male	32 (42.7)	17 (41.5)	15 (44.1)	.82
Female	43 (57.3)	24 (58.5)	19 (55.9)	
**Race and ethnicity**				
White	72 (96.0)	39 (95.1)	33 (97.1)	.41
Black	1 (1.3)	0 (0)	1 (2.9)	
Hispanic	1 (1.3)	1 (2.4)	0 (0)	
Unknown	1 (1.3)	1 (2.4)	0 (0)	
**Age, y**				
14	1 (1.3)	1 (2.4)	0 (0)	.42
15	3 (4.0)	2 (4.9)	1 (2.9)	
16	5 (6.7)	3 (7.3)	2 (5.9)	
17	9 (12.0)	7 (17.1)	2 (5.9)	
18	35 (46.7)	16 (39.0)	20 (58.8)	
19	22 (29.3)	12 (29.3)	9 (26.5)	
Mean age, y (SD)	17.8 (1.1)	17.7 (1.3)	18.0 (0.9)	.30^c^

MTI indicates Motivational Tobacco Intervention; BA, Brief Advice; NA, not applicable.

a Baseline analyses conducted on 75 participants; one participant withdrew from study after baseline analyses.

b *P* values derived from *χ* ^2^ test except where indicated.

c *P* value derived from *t *test for mean values.

**Table 2:** Smoking Status and Baseline Comparisons, by Intervention Group, Emergency Department-based Motivational Teenage Smoking Intervention, Morgantown, WVa, 2002–2004

Items	Total		MTI Group		BA Group		*P* a

	No. Respondents	Mean (SD)	No. Respondents	Mean (SD)	No. Respondents	Mean (SD)	
Average no. days youth smoked in the past 30 days	75	25.4 (8.8)	41	27.0 (7.3)	34	23.5 (10.1)	.09
Average no. cigarettes smoked daily	56	9.9 (8.0)	31	10.2 (7.3)	25	9.5 (8.9)	.75
Average no. cigarettes smoked on weekdays	75	8.5 (6.7)	41	9.3 (6.5)	34	7.4 (6.9)	.23
Average no. cigarettes smoked on weekends	74	12.7 (8.2)	41	14.6 (8.3)	33	10.4 (7.5)	.03
Average no. times tried to quit or cut back	56	2.0 (1.0)	29	2.2 (1.2)	27	1.7 (0.8)	.05
Average nicotine dependence score	75	3.6 (1.8)	41	3.9 (1.7)	34	3.3 (1.8)	.14
Average CO score^b^	67	12.7 (15.1)	35	10.2 (9.4)	32	15.4 (19.3)	.16
**Ever tried to quit or cut back smoking**							
No. who responded yes (%)	56 (74.7)		29 (70.7)		27 (79.4)		.40^c^
No. who responded no (%)	19 (25.3)		12 (29.3)		7 (20.6)		
Total no. respondents (%)	75 (100.0)		41 (100.0)		34 (100.0)		

MTI indicates Motivational Tobacco Intervention; BA, Brief Advice; CO, carbon monoxide; NA, not applicable.

a *P* values derived from *t *test (two-tailed) except where indicated.

b CO <9 ppm was used to validate patients' self-reported nonsmoker status.

c *P* value derived from *χ* ^2^ test (*χ* ^2^ = .74).

**Table 3:** Quit Rates, by Intervention Group and Subsample, Emergency Department-based Motivational Teenage Smoking Intervention, Morgantown, WVa, 2002–2004

Time Elapsed Since Baseline	**MTI Group**		**BA Group**		***P* a**

	No. Respondents	**No. Quitters(%)**	No. Respondents	** No. Quitters(%)**	
**Compliant subsample**					
1 month	11	2 (18.2)	NA	NA	NA
3 months	17	1 (5.9)	NA	NA	NA
6 months	16	1 (6.3)	12	1 (8.3)	.60
**Intent-to-treat subsample**					
1 month	40	2 (5.0)	NA	NA	NA
3 months	40	1 (2.5)	NA	NA	NA
6 months	40	1 (2.5)	34	1 (2.9)	.55

MTI indicates Motivational Tobacco Intervention; BA, Brief Advice;

NA, not applicable because BA group received only one follow-up call at 6 months.

a *P *values derived from *χ* ^2^ test using Yates correction.

**Table 4:** Reduction Rates^a^, by Intervention Group and Subsample, Emergency Department-based Motivational Teenage Smoking Intervention, Morgantown, WVa, 2002–2004

Time Elapsed Since Baseline	MTI Group		BA Group		***P*** **b**	**Effect Size (Cohen's h)**

	No. Respondents	No. Reducers**(%)**	No. Respondents	No. Reducers(%)		
**Compliant subsample**						
1 month	9	4 (44.4)	NA	NA	NA	NA
3 months	16	9 (52.9)	NA	NA	NA	NA
6 months	15	8 (53.3)	11	2 (18.2)	.16	.38
**Intent-to-treat subsample**						
1 month	38	4 (10.5)	NA	NA	NA	NA
3 months	39	9 (23.1)	NA	NA	NA	NA
6 months	39	8 (20.5)	33	2 (6.1)	.15	.15

MTI indicates Motivational Tobacco Intervention; BA, Brief Advice;

NA, not applicable because BA group received only one follow-up call at 6 months.

a Quitters were excluded from reduction rate calculations (numerator and denominator).

b *P *values derived from *χ* ^2^ test using Yates correction.

**Table 5:** Mean Percentage Reduction Rates Among Teenagers who Reduced Smoking, by Intervention Group, Emergency Department-based Motivational Teenage Smoking Intervention, Morgantown, WVa, 2002–2004

**Time Elapsed Since Baseline**	MTI Group		BA Group		*P* a	**Effect Size (Cohen's h)**

	No. Respondents	**Mean (SD)**	No. Respondents	**Mean (SD)**		
1 month	4	54.6 (31.6)	NA	NA	NA	NA
3 months	9	42.5 (32.4)	NA	NA	NA	NA
6 months	8	31.4 (18.9)	2	22.1 (1.0)	.53	.69

MTI indicates Motivational Tobacco Intervention; BA, Brief Advice

NA, not applicable because BA group received only one follow-up call at 6 months.

a *P* value derived from *t *test (two-tailed).

## References

[1] Sussman S, Lichtman K, Ritt A, Pallonen UE (1999). Effects of thirty-four adolescent tobacco use cessation and prevention trials on regular users of tobacco products. Subst Use Misuse.

[2] Sussman S (2002). Effects of sixty six adolescent tobacco use cessation trials and seventeen prospective studies of self-initiated quitting. Tobacco Induced Diseases. Tobacco Induced Diseases.

[3] Horn K, Dino G, Goldcamp J, Kalsekar I, Mody R (2005). The impact of Not On Tobacco on teen smoking cessation: end-of-program evaluation results, 1998-2003. J Adoles Res.

[4] Sussman S, Dent CW, Lichtman KL (2001). Project EX: outcomes of a teen smoking cessation program. Addict Behav.

[5] Mermelstein R (2003). Teen smoking cessation. Tob Control.

[6] Miller WR, Rollnick S (2002). Motivational interviewing: preparing people for change.

[7] Miller WR, Sanchez VC (1993). Motivating young adults for treatment and lifestyle change.

[8] Graham AW, Fleming MS (1998). Brief interventions.

[9] Lawendowski LA (1998). A motivational intervention for adolescent smokers. Prev Med.

[10] Colby SM, Monti PM, Barnett NP, Rohsenow DJ, Weissman K, Spirito A (1998). Brief motivational interviewing in a hospital setting for adolescent smoking: a preliminary study. J Consult Clin Psychol.

[11] Fleming MF, Barry KL, Manwell LB, Johnson K, London R (1997). Brief physician advice for problem alcohol drinkers. A randomized controlled trial in community-based primary care practices. JAMA.

[12] McCambridge J, Strang J (2005). Deterioration over time in effect of Motivational Interviewing in reducing drug consumption and related risk among young people. Addiction.

[13] Aubrey LL (1998). Motivational interviewing with adolescents presenting for outpatient substance abuse treatment [unpublished doctoral dissertation].

[14] Carroll KM, Connors GJ, Cooney NL, DiClemente CC, Donovan DM, Kadden RR (1998). Internal validity of Project MATCH treatments: discriminability and integrity. J Consult Clin Psychol.

[15] Horn K, Dino G (2001). The Power Guide: a self-help audio workbook for teen smokers.

[16] WVU HealthLine.

[17] Pomerleau CS, Carton SM, Lutzke ML, Flessland KA, Pomerleau OF (1994). Reliability of the Fagerstrom Tolerance Questionnaire and the Fagerstrom Test for Nicotine Dependence. Addict Behav.

[18] Horn K, Dino G, Gao X, Momani A (1999). Feasibility evaluation of Not On Tobacco: The American Lung Association's new stop smoking program for adolescents. Health Educ.

[19] Dino G, Horn K, Goldcamp J, Fernandes A, Kalsekar I (2001). A 2-year efficacy study of Not On Tobacco in Florida: an overview of program successes in changing teen smoking behavior. Prev Med.

[20] Sussman S, Dent CW, Severson H, Burton D, Flay BR (1998). Self-initiated quitting among adolescent smokers. Prev Med.

[21] Velicer WF, Prochaska JO, Rossi JS, Snow MG (1992). Assessing outcome in smoking cessation studies. Psychol Bull.

[22] Lichtenstein E, Glasgow RE (1992). Smoking cessation: what have we learned over the past decade?. J Consult Clin Psychol.

[23] Horn D, Waingrow S (1967). Smoking behavior change.

[24] Gottlieb NH (1983). The determination of smoking types: Evidence for a sociological-pharmacological continuum. Addict Behav.

[25] Tate JC, Stanton AL (1990). Assessment of the validity of the Reasons for Smoking scale. Addict Behav.

[26] Lipkin M (1990). Functional status measurement in primary care.

[27] Rollnick S, Butler CC, Stott N (1997). Helping smokers make decisions: the enhancement of brief intervention for general medical practice. Patient Educ Couns.

[28] Dino GA, Horn KA, Zedosky L, Monaco K (1998). A positive response to teen smoking: why N-O-T?. NASP Bulletin.

[29] Perez-Stable EJ, Sabogal F, Marin G, Marin BV, Otero-Sabogal R (1991). Evaluation of "Guia para dejar de fumar," a self-help guide in Spanish to quit smoking. Public Health Rep.

[30] Schorling JB (1995). The stages of change of rural African-American smokers. Am J Prev Med.

[31] Orleans CT, Boyd NR, Bingler R, Sutton C, Fairclough D, Heller D (1998). A self-help intervention for African American smokers: tailoring cancer information service counseling for a special population. Prev Med.

[32] DiGiusto E (1994). Pros and cons of cessation interventions for adolescent smokers at school.

[33] Keppel G (1991). Design and analysis: a researcher's handbook.

[34] Woolard R, Monti PM,  Colby S, Barnett N, Spirito A, Rohsenow DJ (1999). Brief motivational intervention for older adolescents with alcohol related emergency department visits. Acad Emerg Med.

[35] Monti PM, Colby SM, Barnett NP, Spirito A, Rohsenow DJ, Myers M (1999). Brief intervention for harm reduction with alcohol-positive older adolescents in a hospital emergency department. J Consult Clin Psychol.

[36] Spirito A, Monti PM, Barnett NP, Colby SM, Sindelar H, Rohsenow DJ (2004). A randomized clinical trial of a brief motivational intervention for alcohol-positive adolescents treated in an emergency department. J Pediatr.

[37] O'Leary-Tevyaw T, Monti PM (2004). Motivational enhancement and other brief interventions for adolescent substance abuse: foundations, applications and evaluations. Addiction.

[38] Hatsukami DK, Lemmonds C, Zhang Y, Murphy SE, Le C, Carmella SG (2004). Evaluation of carcinogen exposure in people who used "reduced exposure" tobacco products. J Natl Cancer Inst.

[39] Miller WR, Mount KA (2001). A small study of training in motivational interviewing: does one workshop change clinician and client behavior?. Behavioural and Cognitive Psychotherapy.

[40] Miller WR (1995). Increasing motivation for change.

